# Graphene oxide–chloroquine conjugate induces DNA damage in A549 lung cancer cells through autophagy modulation

**DOI:** 10.3762/bjnano.16.24

**Published:** 2025-03-03

**Authors:** Braham Dutt Arya, Sandeep Mittal, Prachi Joshi, Alok Kumar Pandey, Jaime E Ramirez-Vick, Govind Gupta, Surinder P Singh

**Affiliations:** 1 CSIR-National Physical Laboratory, Dr K. S. Krishanan Marg, New Delhi-12, India; 2 Academy of Scientific & Innovative Research (AcSIR), New Delhi-20, India; 3 Department of Higher Education, Shiksha Sadan, Sector-5, Panchkula-134114, India; 4 CSIR-Indian Institute of Toxicology Research (CSIR-IITR), 31, Mahatma Gandhi Marg, Lucknow-226001, India; 5 Department of Biomedical, Industrial & Human Factors Engineering, Wright State University, Dayton, Ohio 45435, United States

**Keywords:** A549 cells, autophagy, chloroquine, DNA damage, graphene oxide, nanoconjugates, SQSTM1/p62

## Abstract

Autophagy is a highly regulated catabolic process by which unnecessary, dysfunctional, or damaged proteins and other cellular components are degraded and recycled to promote cellular differentiation, survival, and development. In response to endogenous or exogenous stresses, cancer cells use autophagy pathways for survival through activation of complex DNA damage repair (DDR) mechanisms. In the present study, we demonstrated the genotoxicity induced in A549 lung cancer cells by exposure to the GO–Chl nanoconjugate and elucidated the role of autophagy modulation in harnessing the DNA-damage response. GO–Chl causes loss of plasma membrane integrity, cell cycle arrest, and significant genotoxicity in A549 cells. Further, elevated expression of key autophagy proteins beclin-1, ATG-7, LC-3-I/II, and SQSTM1/p62 reveal that inhibition of autophagy plays a crucial role in regulating DDR capabilities of cancer cells. The results indicate that the interplay between DDR and autophagy pathways may open new paradigms for developing effective combinatorial nanoscale drug systems against multidrug-resistance cancers.

## Introduction

Despite of advances in basic and clinical research, an increased mortality rate is seen worldwide in cancer-associated deaths [1]. The heterogeneous and complex tumor microenvironment along with intrinsic and/or acquired drug resistance mechanisms, such as increased drug efflux, DNA damage repair, and activation of pro-survival cell signaling cascades, alterations in drug target moieties limit the effectiveness of chemotherapeutic treatments [2–3]. In general, chemotherapeutic drugs inhibit the cancer progression and metastases by directly or indirectly targeting DNA of cancer cells, inducing a variety of DNA lesions. Cancer cells are equipped with complex molecular signaling pathways for recognition and repair of damaged DNA [4]. The activation of the DNA-damage response (DDR) machinery by phosphatidylinositol 3-kinase-related kinase (PIKKs) family proteins, such as ataxia-telangiectasia mutated, Rad3-related (ATR), and DNA-dependent protein kinase catalytic subunit (DNA-PKcs), upon exposure to chemotherapeutics is a major hurdle in the treatment of chemoresistant tumors due to its complexity and redundancy [5]. Various preclinical studies have shown that inhibition of DDR either through autophagy modulation or poly (ADP-ribose) polymerase (PARP) inhibition could provide a better therapeutic response [6–7].

Recently, nanomedicine has shown immense potential/efficacy in the treatment of chemoresistant tumors by providing improved molecular targeting, better pharmacokinetics, and reduced side effects. Nanomaterials can directly target DNA or inhibit the DDR and sensitize cancer cells to chemotherapeutics in multidrug resistant tumors [8–10]. Satapathay reported DNA damage and apoptotic cell death in HCT116 cells, human colorectal epithelial carcinoma cells, after exposure to starch-capped silver nanoparticles (AgNPs) [11]. Gemcitabine-encapsulated poly(lactic-*co*-glycolic acid) (PLGA) nanoparticles have been shown to enhance cell death in chemoresistant PANC1 cells, human pancreatic epithelial carcinoma cells [12]. Also, TiO_2_ nanoparticles can sensitize A549 cells, human lung epithelial carcinoma cells towards the genotoxic agent methyl methanesulphonate through disruption of the DDR process [13]. Recently, ZnO nanoparticles induced significant cytotoxicity and DNA double-strand breaks in SKOV3 cells, human ovarian epithelial cancer cells, through induction of oxidative stress and autophagy modulation [14].

Graphene oxide (GO) due to its unique physicochemical properties has attracted vast scientific attention as an efficient drug delivery carrier and modulator of biological activities, including autophagy, DDR, and intracellular transportation of therapeutics into cancer cells [15–17]. Exposure to GO has been shown to trigger autophagy response through toll-like receptors in CT26 cells, mouse colorectal fibroblast carcinoma cells, and lysosomal destabilization in PC12 cells, rat adrenal pheochromocytoma cancer cells, leading to cell death [18–19]. Graphene oxide has been shown to sensitize CT26 (mouse colorectal fibroblast carcinoma cells), Skov-3 (human ovarian epithelial cancer cells), HeLa (human cervical epithelial adenocarcinoma cells), and Tramp-C1 (mouse prostate epithelial adenocarcinoma) cancer cells to chemotherapeutics through enhanced acetylation of histone in the nucleus causing increased decondensing of chromatin, making cancer cells more susceptible to DNA damage [15]. Graphene oxide has also been shown to selectively target cancer stem cells among multiple cell types by inhibiting a number of different signal transduction pathways, including WNT, Notch, STAT 1/3, and NRF-2, respectively [20]. Graphene oxide nanosheets have been shown to selectively disrupt the cell membrane and cytoskeleton of cancer cells through activation of FAK-Rho-ROCK pathway and suppressed expression of integrin [21]. It has also been found that nuclear accumulation of p62, due to inhibition of autophagy in cancer cells, subsequently suppresses chromatin and histone ubiquitination and inhibits the DDR mechanism [22]. Cancer cells are known to employ autophagy in response to DNA damage as a survival mechanism, and inhibition of autophagy could be an excellent treatment modality in multidrug resistant tumors [23–24]. Recently, it has also been found that chloroquine- (Chl, autophagy inhibitor) conjugated GO induces necroptotic cell death in A549 cells through accumulation of p62 mediated by altered autophagic flux, reactive oxygen species (ROS) level, and activation of RIPK1 [25]. In the present study, we investigated the DNA-damage-mediated cell death mechanism in A549 cells upon exposure to a graphene oxide–chloroquine (GO–Chl) nanoconjugate. Our results have shown that exposure of GO–Chl nanoconjugate induced DNA fragmentation/damage in A549 cells, causing significant genotoxicity which ultimately leads to enhanced cancer cell death [25]. Further, we have analyzed the meaningful partnership between autophagy modulation and DNA damage in GO–Chl exposure A549 cells. Furthermore, immunoblot analysis of autophagy biomarkers reveals the relationship between autophagy and DNA damage in response to GO–Chl exposure in human lung epithelial carcinoma (A549) cells.

## Experimental

### Materials

Graphite powder (Cat. No. 282863), chloroquine (Cat. No. C6628), paraformaldehyde (PFA; Cat. No. 24-0630), dimethyl sulfoxide (DMSO; Cat. No. D2650), propidium iodide (Cat. No. P4170), Triton X-100 (Cat. No. T8787), monodansylcadaverine (MDC; Cat. No. D4008), anti-SQSTM1 (Cat. No. P0067), anti-MLKL primary antibody (Cat. No. SAB5700808), protein A/G agarose beads (Cat. No. IP10), and hydrogen peroxide (H_2_O_2_; Cat. No. H1009) were purchased from Sigma-Aldrich (St. Louis, MO, USA). Phosphate-buffered saline (Ca^+2^, Mg^+2^ free; PBS; Cat. No. 14200166), Dulbecco’s modified Eagle’s medium: nutrient mixture F-12 (Ham) (1:1) powder (DMEM F-12; Cat. No. 11320033), trypsin–EDTA (Cat. No. 25200056), fetal bovine serum (FBS; Cat. No. A5256701), antibiotic and antimycotic solution (100X; 10,000 U/mL penicillin, 10 mg/mL streptomycin, 25 μg/mL amphotericin-B; Cat No. 15240062), mouse and rabbit secondary antibodies conjugated to HRP, 4',6-diamidino-2-phenylindole (DAPI; Cat No. 62248) were purchased from Life Technologies (Invitrogen, Carlsbad, CA, USA). A549 human lung adenocarcinoma cells were obtained from American Type Cell Culture (Cat. No. CCL-185; ATCC, Manassas, VA, USA). Primary antibodies anti-β-actin (Cat. No. 4970), anti-LC-3-I/II (Cat. No. 12741), anti-ATG-7 (Cat. No. 88577) and anti-beclin-1 (Cat. No. 3495) were obtained from Cell Signaling Technology (Danvers, MA, USA). The GFP-LC3 plasmid was a kind gift from Dr. Soumya Sinha Roy, CSIR-IGIB, India. The antifade mounting media Vectashield was purchased from Vector Laboratories (Burlingame, CA, USA). All other chemicals were locally obtained and were of analytical reagent grade. Cell culture plastic wares were obtained from Thermo Scientific Nunc (Rochester, New York).

### Synthesis of graphene oxide

**Figure 1:**
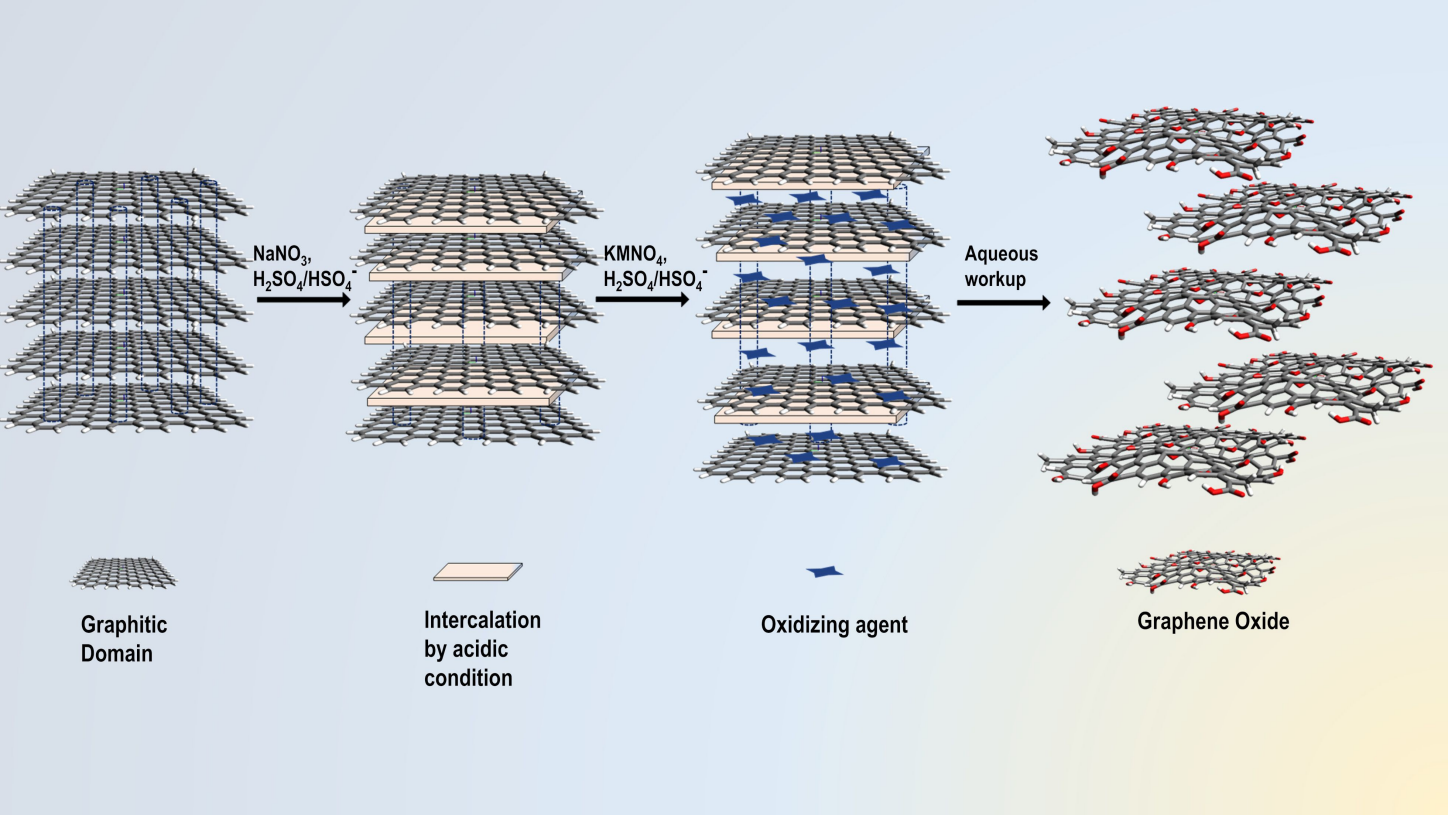
Schematic representation for the process of chemical exfoliation and oxidation of graphite powder to obtain highly exfoliated graphene oxide nanosheets. (Figure 1 was created by the authors using Avogadro: an open-source molecular builder and visualization tool. Version 1.0.2n http://avogadro.cc/) [40].

Highly exfoliated GO nanosheets were chemically synthesized using slight modifications to the method documented by Dimiev et al. [26–27]. Briefly, 0.5 g of graphite powder was treated with a mixture of 0.5 g of NaNO_3_ and 40 mL of H_2_SO_4_ (98%) which intercalates between graphitic layers. Finely powdered KMnO_4_ (1.5 g) was slowly added (time span ≈30 min) to the reaction mixture under continuous stirring at 4 °C in an ice bath (0–5 °C). The reaction mixture was kept under vigorous stirring for 12 h at room temperature, until a dark brownish precipitate appeared, followed by addition of 1.5 mL of H_2_O_2_ (30%) and 100 mL of ice-cold ultrapure water to stop the oxidation reaction and eliminate unreacted KMnO_4_. The chemistry involved in the chemical exfoliation of graphite is shown in Figure 1. The purified graphitic sheets were collected using successive centrifugation steps at 13,000 rpm for 30 min and washing with deionized (DI) water several times, until the hydrolysis of covalent sulfates formed during oxidation [26–27]. The final product of exfoliated GO nanosheets were extracted through freeze-drying.

### Binding of chloroquine onto graphene oxide nanosheets

Chloroquine molecules were bound to GO nanosheets through noncovalent π–π interactions between the quinoline of Chl and the graphitic domain of GO, according to a previously reported procedure [25]. Briefly, 100 mL of an aqueous dispersion of GO (500 μg/mL) nanosheets was mixed with 15 mL of an aqueous solution of Chl diphosphate (250 μg/mL) under continuous stirring at room temperature in the dark for 24 h. The final GO–Chl nanoconjugate was collected by centrifugation at 9500 rpm for 15 min followed by freeze-drying. The supernatant was separated for quantitative estimation of the unbound drug.

### Physical characterization techniques

The functional group and structural analysis of GO and GO–Chl nanoconjugate were studied using a Fourier-transform infrared spectrometer (Cary 630 FTIR, Agilent, CA, USA) in ATR mode and a Raman spectrophotometer (Ranishaw win-via reflex spectrometer, Tokyo, Japan) with a 514 nm Nd:Yag laser as the excitation source. The optical properties were measured employing a UV–vis spectrophotometer (Cary 5000 UV-VIS-NIR, Agilent, CA, USA) in the 200–800 nm range. The morphology of GO nanosheets was analyzed employing a high-resolution transmission electron microscope (Technai G2 F30 STWIN, Japan), field emission scanning electron microscope (FEI, Quanta FEG 450, USA), and atomic force microscope (Nanoscope, Veeco V, USA) [28–30].

### Cell culture

A549 cells from the American Type Culture Collection (ATCC, Manassas, VA, USA) were cultured using DMEM F-12 medium (Life Technologies, Invitrogen, Carlsbad, CA, USA), supplemented with 10% heat-inactivated fetal bovine serum, 0.2% sodium bicarbonate, and 10 mL/L of antibiotic and antimycotic solution. The cells were maintained at 37 °C under a humidified atmosphere of 5% CO_2_. The cells were seeded onto 96-well and 12-well plates, and onto 75 cm^2^ culture flasks, depending on the experiment, and were grown overnight. Then, fresh medium with varying concentrations (1–100 μg/mL) of GO–Chl nanoconjugates was added to the cells and incubated for a time period, which depended on the specific experiment. Each group contained four technical replicates and three biological replicates for each experiment. In each assay cells without nanoconjugates were used as a control.

### Transmission electron microscopy analysis

The A549 cells (2 × 10^5^ cells/mL/well in 6-well plates) were exposed to 25 μg/mL of GO–Chl for 24 h and washed with 1× PBS to remove any GO–Chl nanoconjugates not uptaken by the cells. Cells were then harvested using trypsin–EDTA, washed with 1× PBS followed by fixation with 2.5% glutaraldehyde for 4 h. Then, fixed cells were washed using 0.1 M of sodium cacodylate buffer, post-fixed in 1% osmium tetraoxide for 4 h at 4 °C, and again washed with 0.1 M sodium cacodylate buffer. After that, cells were dehydrated using different percentages of acetone series (15–100%) and incubated with an AralditeR–DDSA mixture overnight at room temperature. The cell blocks were made by embedding the samples in pure resin followed by curing at 60 °C for 24 h. The blocks were then subjected to a Leica UC7 ultramicrotome (Wetzlar, Germany) to make ultrathin (60 nm) sections followed by staining with uranyl acetate and lead citrate. The sections were allowed to air dry before analysis using the TecnaiTM G2 Spirit (FEI Company, Eindhoven, Netherlands) instrument at an accelerating voltage of 80 kV equipped with a Gatan camera.

### Propidium iodide uptake analysis

Propidium iodide (PI), a positively charged nucleic acid dye, specifically exhibits fluorescence after binding with DNA of cells with compromised membrane and used for quantitative estimation of plasma membrane integrity using flow cytometry [31]. Briefly, A549 cells (1 × 10^5^ cells/mL/well) were seeded onto 12-well culture plates and exposed to varying concentrations (1–100 μg/mL) of GO–Chl for 24 h. Cells were washed with 1× PBS, harvested using 0.25% trypsin, and centrifuged at 1000 rpm for 10 min to remove excess GO–Chl nanoconjugates. Furthermore, cells were resuspended in 100 μL of 1× PBS and incubated with the PI dye (stock: 1 mg/mL; working solution: 2 μL/100 μL) for 10 min at room temperature in the dark. Further, the cell suspension was diluted by adding 400 μL of 1× PBS, and red fluorescence emitted from PI was analyzed by flow cytometry (BD FACS Canto II, BD Biosciences, San Jose, CA, USA) using a 650 ± 13 nm band-pass filter. Three independent experiments were performed for each group. The proportion of cells with compromised membrane integrity was analyzed using the FACS Diva software (version 6.1.2, BD Biosciences, San Jose, CA, USA). Values represent mean ± standard error (SE) of three independent experiments. A value of *p* < 0.05 (*) was considered statistically significant.

### Cell cycle analysis

The cell cycle analysis was carried out by flow cytometry using PI [31]. Briefly, A549 cells were treated with different concentrations (1–100 μg/mL) of the GO–Chl nanoconjugate for 24 h. The cells were washed twice with cold 1× PBS, harvested using 0.25% trypsin, centrifuged at 1000 rpm for 10 min, and the pellet was resuspended in 300 μL of 1× PBS. Furthermore, the cells were fixed with 70% ice-cold ethanol and incubated overnight at −20 °C. Thereafter, cells were centrifuged at 1200 rpm for 4 min and the resulting pellet was resuspended in 200 μL of lysis buffer (1× PBS with 0.2% Triton X-100) and incubated at 4 °C for 30 min. Lysed cells were then treated with RNase (10 mg/mL) for 30 min at 37 °C to eliminate RNA as PI can binds to double-stranded RNA. Finally, cells were once again centrifuged at 1200 rpm for 10 min and the pellet was resuspended in 500 μL of 1× PBS containing 10 μL of PI dye (1 mg/mL) and stored at 4 °C until analysis using flow cytometry. Three independent experiments were performed for each group. Values represent mean ± SE of three independent experiments. A value of *p* < 0.05 (*) was considered statistically significant.

### Comet assay/single-cell gel electrophoresis for DNA damage analysis upon exposure of A549 cells to GO–Chl nanoconjugates

Single-cell gel electrophoresis was used to investigate DNA denaturation and single-strand DNA breaks upon exposure of A549 lung cancer cells to GO–Chl nanoconjugates. For quantitative estimation of DNA damage, we followed the method developed by Singh et al. [32] and base slides were prepared according to the method of Bajpayee [33].

Briefly, A549 cells (1 × 10^5^ cells/mL/well) were seeded onto 12-well culture plates and exposed to varying concentrations (1–100 μg/mL) of GO–Chl for 6 h. Cells were washed twice with 1× PBS to remove any GO–Chl nanoconjugate not uptaken by the cells. Cells were then harvested using trypsin–EDTA and resuspended in 100 μL of 1× PBS, followed by mixing with 1% low-melting-point agarose (LMPA, prepared in 1× PBS) to achieve a final concentration of 0.5%. Thereafter, 80 μL of the suspension was layered onto base slides (pre coated with 1% normal-melting agarose; NMA), evenly spread with a coverslip, and kept on ice to allow gelation. The coverslip was carefully removed followed by the addition of a third layer of 90 μL of 0.5% LMPA, carefully spreading with a coverslip and kept on ice to allow gelation.

Duplicate slides for each sample were prepared and were kept in freshly prepared and chilled lysis solution (146.1 g NaCl, 37.2 g EDTA, 1.2 g Tris, pH 10 with 1% Triton X 100 added just before use) at 4 °C overnight. Further, the slides were placed in a horizontal gel electrophoresis tank containing freshly prepared chilled electrophoresis solution (1 mM of EDTA, 300 mM of NaOH, pH > 13) for 20 min for DNA unwinding and subsequently subjected to electrophoresis with a 300 mA current, 0.7 V/cm at 4 °C under dimmed light for 30 min. After electrophoresis, the slides were treated with Tris buffer (0.4 M, pH 7.5) 3× at 5 min per cycle to neutralize excess alkaline solution, stained with 75 μL of ethidium bromide (20 µg/mL), and stored in a humidified slide box until scoring. The slides were scored using a fluorescent microscope (DMLB, Leica, Germany) coupled with a CCD camera and an image analysis system (Andor Technology, Belfast, UK) and a software (KOMET 5.0, Kinetic Imaging, UK) at 400× magnification. Three independent experiments were performed for each group. The mean value of three Comet parameters, tail DNA (%), tail length (μm), and Olive tail moment (OTM) were considered during the analysis. Values represent mean ± SE of three independent experiments. A value of *p* < 0.05 (*) was considered as statistically significant.

### Autophagy analysis

The effect of GO–Chl exposure on autophagy modulation in A549 cells was studied employing the following assays:

### Fluorescent monodansylcadaverine staining

Fluorescent monodansylcadaverine selectively accumulates in acidic vacuoles and has been used as a tracer for autophagic vacuoles [34]. Briefly, A549 cells were plated onto 20 mm round glass coverslips and allowed to adhere overnight. On the next day, cells were exposed to 25 μg/mL of the GO–Chl nanoconjugate (dose chosen based on cell death analysis in our previous study) [25] for 24 h, rinsed with 1× PBS, and stained with 50 mM of MDC at 37 °C for 1 h. Finally, cells were washed with 1× PBS, and the cellular fluorescence changes were observed using a Nikon Eclipse Ti-S inverted fluorescent microscope equipped with a Nikon Digital slight Ds-Ri1 CCD camera and a NIS element BR imaging software (Nikon, Minato Tokyo, Japan). Three independent experiments were performed for each group and a representative image is shown in the results.

### Transfection of the GFP-LC3 plasmid

The GFP-LC3 plasmids (Gene Insert: MAP1LC3B; kind gift from Dr. Soumya Sinha Roy, CSIR - IGIB, India) were employed to evaluate the quantitative formation of autophagic puncta upon GO–Chl exposure [35]. Briefly, A549 cells were plated onto 4-well chamber slides and transiently co-transfected with the mammalian GFP-LC3 plasmid employing the conventional lipid-mediated gene delivery reagent lipofectamine 2000 following manufacturer’s instructions. After 6 h, the transfection medium was replaced with fresh medium and the cells were incubated overnight. The cells were then exposed to 25 μg/mL GO–Chl for 24 h and washed with 1× PBS. The cells were then fixed in 4% paraformaldehyde at 4 °C for 30 min, counterstained with DAPI for nuclear staining, mounted using antifade, and analyzed using confocal microscopy.

### Immunoblot analysis

The expression level of various autophagy-related proteins was analyzed using immunoblotting to investigate the autophagy process. Briefly, A549 cells (5 × 10^5^ cells/flask) were seeded onto a 25 cm^2^ culture flask and exposed to different concentrations (1–100 μg/mL) of the GO–Chl nanoconjugate for the desired time. The cells were washed twice with cold 1× PBS, harvested, and the whole-cell extract was prepared using a radioimmunoprecipitation assay (RIPA) cell lytic reagent (Cat. No. R0278; Sigma-Aldrich, St. Louis, Missouri, USA) supplemented with protease and phosphatase inhibitors. The protein content was quantitated using the Bradford assay and further resolved by sodium dodecyl sulfate polyacrylamide gel electrophoresis (SDS-PAGE) [36], transferred to a polyvinylidene difluoride (PVDF) membrane by electroblotting, blocked with casein-blocking buffer (Sigma-Aldrich) for 1 h at room temperature, and incubated with primary antibodies in 1× TBST overnight at 4 °C. On the next day, the blots were washed with 1× TBST and incubated with the corresponding horseradish peroxidase-coupled anti-rabbit or anti-mouse secondary antibodies (Cell Signaling Technology, MA, USA). The proteins were visualized with Super Signal West Femto reagents (Pierce Biotechnology, Rockford, IL, USA) through chemiluminescence. Three independent experiments were performed for each group. The values represent the mean ± SE of three independent experiments. A value of *p* < 0.05 (*) was considered statistically significant.

## Results and Discussion

### Physical characterization analysis

The synthesis of graphene oxide involves chemical exfoliation and oxidation of graphite powder. We employed a method with slight modifications and optimizations to the original documented method by Dimiev et al. [26–27] as shown in Figure 1. The chemical synthesis of highly pure graphene oxide nanosheets depends on several factors such as the source of graphite, the weight equivalent of KMnO_4_, reaction time, and washing conditions [26,37]. Most importantly, the degree of oxidation of graphene oxide plays a crucial role in controlling the cytotoxicity of the material [38–39]. To achieve higher oxidation, six times weight equivalent of KMnO_4_ (added in two steps of 3 equiv each) were used relative to the graphite powder used. Chloroquine binds to the surface of graphene oxide nanosheets through noncovalent π–π interactions between the quinoline ring of Chl and the sp^2^ hybrid π-bonded carbon framework of graphene oxide.

The formation of GO–Chl nanoconjugates was confirmed by investigating the optical, functional, structural, and morphological properties employing standard analytical characterization techniques. The optical properties of GO and GO–Chl were assessed using UV–vis spectroscopy (Supporting Information File 1). Supporting Information File 1, Figure S1a, reveals the appearance of characteristic bands around 230 nm and 295 nm for GO which corresponds to π–π* and n–π* electronic transitions, respectively. The observed high intensity π–π* plasmon peak around 230 nm is attributed to well-defined nanoscale sp^2^ hybrid π-bonded networks and chromophore aggregation due to the presence of C=C, C=O, and C–O bonds [41]. The presence of a shoulder band around 295 nm corresponds to the well-defined n–π* electronic transitions due to the presence of C=O functional groups on the surface of GO. The appearance of the Chl characteristic band at around 343 nm, in GO–Chl, could be related to the binding of Chl to GO [42].

The functional groups in GO and GO–Chl nanoconjugates were analyzed by FTIR spectroscopy. Supporting Information File 1, Figure S1b shows the FTIR spectrum of GO, Chl, and GO–Chl in the range of 500–3800 cm^−1^, respectively. The presence of a broad peak around 3200–3400 cm^−1^ and other characteristic bands around 1720 cm^−1^, 1620 cm^−1^, 1150 cm^−1^, and 1050 cm^−1^ for GO, correspond to the stretching vibration of –OH, C=O, C=C, C–OH, and C(O)C bonds, respectively [26,43]. The appearance of a peak around 2980 cm^−1^ in both Chl and GO–Chl corresponds to the C–H stretch of the methyl group present in Chl and GO–Chl, respectively. Furthermore, the presence of C–N stretch (1360 cm^−1^), N–H bending of amine, and C–Cl stretch (530 cm^−1^) in Chl and GO–Chl reveals the formation of the GO–Chl nanoconjugate [25,44].

Further, Raman spectroscopy was utilized to evaluate the formation of GO (after the oxidation of graphite powder) and the GO–Chl nanoconjugate. The structural properties of graphite and GO nanosheets were investigated through the comprehensive analysis of the characteristic graphitic domain band (G band) and defect band (D band) in the Raman spectra (recorded at an excitation wavelength of 514 nm) of the compounds. Supporting Information File 1, Figure S1c reveals the appearance of the G band (1581 cm^−1^) and D band (1352 cm^−1^) for graphite, corresponding to the E_2g_ symmetric vibrations associated with the sp^2^ carbon domain and structural disorders raised due to various factors, respectively [45–46]. Further, the presence of a well-defined D band in the Raman spectra of GO could be attributed to the chemical conversion of graphitic sp^2^ carbon into oxygen-rich functional groups such as C=O, C–OH, and C(O)C [45]. These oxygen-rich functional groups constitute the formation of various structural defects and attributes to the appearance of a relative high-intensity D band in GO. On the other hand, a blue shift in the position of the G band (1581 cm^−1^ to 1596 cm^−1^) was also observed for GO, which could be attributed to increased graphitic amorphization [45]. Finally, the change in the *I*_D_/*I*_G_ ratio for graphite (0.065) and GO (0.929) corresponds to the number of defects relative to the sp^2^ hybrid honeycombed graphitic domain in each compound, respectively. On the other hand, a decrease in the *I*_D_/*I*_G_ ratio (0.882) is observed for the GO–Chl sample compared to that of GO (0.929). This indicates a possible conversion of the sp^3^ carbon to sp^2^ carbon due to the reducing environment imparted by the amino functionalities present on the Chl, which was also corroborated by FTIR data [47]. Furthermore, the decrease in *I*_D_/*I*_G_ corroborates with the bathochromic shift of the π–π* electronic transition in GO–Chl due to the preservation of a sp^2^ carbon framework.

**Figure 2:**
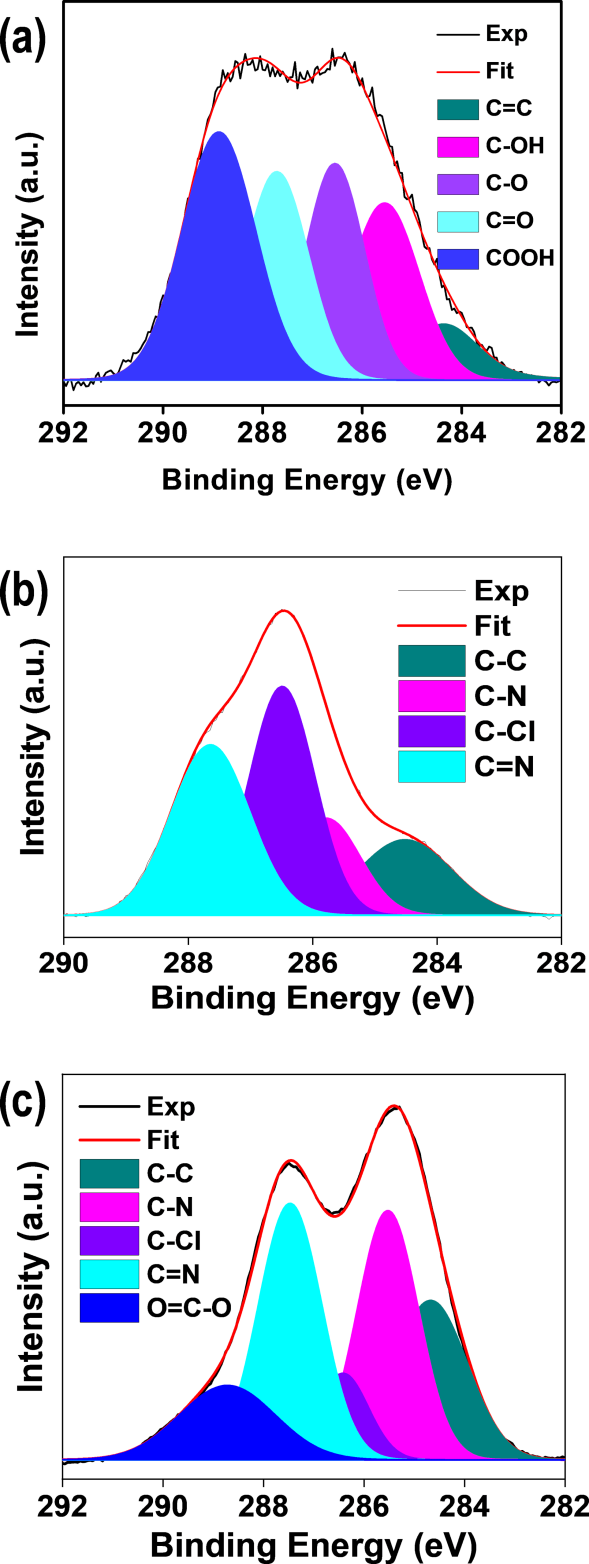
X-ray photoelectron spectroscopy analysis. C 1s core level spectra for GO (a), Chl (b), and GO–Chl (c), respectively. Experimental (black line), fitted data (red line), and deconvoluted fitting components (multicolored regions) are also shown here.

Furthermore, the chemical states/structures of GO, Chl, and GO–Chl were investigated through X-ray photoelectron spectroscopy. Figure 2a–c corresponds to the deconvoluted C (1s) core level of GO, Chl, and GO–Chl respectively. To analyze the relative content of functional groups, the C (1s) peaks of the samples were deconvoluted into different components viz. C=C (284.4), C–C (284.6), C–N (285.6), C–OH (285.6), C–Cl (286.4), C–O (286.6), C=N (287.6), C=O (287.7), and COOH (288.8) eV, respectively, using the Voigt (mixed Gaussian–Lorentzian) function. The peak fitting was performed with respect to the chemical bonding of the materials, the least count of the instrument, and in agreement with the available literature [48–49]. The appearance of high-intensity functional group peaks (286–290 eV) relative to C=C sp^2^ framework peak (graphitic domains, 284.4 eV) in GO indicates the formation of highly exfoliated and oxidized GO nanosheets [48]. The deconvoluted C (1s) core level peaks of GO with the relative intensity of 30.25 % (COOH), 21.54% (C=O), 20.83% (C–O), and 20.09% (C–OH) in comparison to 7.27% (C=C) confirms the high content of oxygen-containing functional group on the surface of GO nanosheets. In contrast, the deconvoluted C (1s) core level of GO–Chl reveals the presence of GO and Chl associated C=C, C–N, C=N, C–Cl and COOH functional groups in the GO–Chl nanoconjugate. The observed reduction in the intensity of COOH peaks for GO–Chl as compared to that of GO, could be attributed to the possible reduction of oxygen-rich functional groups as a result of their interaction with amino groups present in Chl [47]. It was observed that the GO–Chl sample shows the prevalence of C–N and C=N bonds, while C–Cl and C=N bonds were more dominant in Chl. These observations are in good agreement with our FTIR-based analysis. Also, the presence of C=N peaks in Chl and GO–Chl indicates the presence of amino domains of Chl onto GO–Chl nanoconjugates. The observed data with relative % are shown in Supporting Information File 1, Table S1.

The morphological analysis of GO was carried out using field-emission scanning electron microscopy (FESEM) and high-resolution transmission electron microscopy (HRTEM). In Supporting Information File 1, Figure S1d, the FESEM image reveals a well-defined interlocked 3D network of GO nanosheets, with the transparency observed attributed to the formation of single or few layered GO nanosheets [25,50]. In Supporting Information File 1, Figure S1e, the HRTEM micrograph reveals highly transparent GO nanosheets with few wrinkles and folds and corroborates the FESEM observation for the formation of single or few layered GO nanosheets [51]. The selected area of the electron diffraction pattern of GO shown in Supporting Information File 1, Figure S1f, leads to the observation of a six-fold clear diffraction spot pattern, characteristic of a hexagonal crystalline lattice, indicating that GO is not completely amorphous. In addition, the absence of any additional spots other than graphitic structures suggests that oxygen-containing functional groups in GO do not contribute to form-ordered lattice arrangements [28,51]. Further, the morphology and topography of GO nanosheets were analyzed employing atomic force microscopy. Supporting Information File 1, Figure S2 reveals the appearance of few layered interlocked GO nanosheets, and the topographical analysis reveals the thickness in the range of 0.6 to 1.06 nm which could suggest the formation of nearly mono- or few layers of GO nanosheets. These spectroscopic and morphological results were in good agreement with our previous observations [25].

### Quantitative estimation of Chl binding onto GO nanosheets

**Figure 3:**
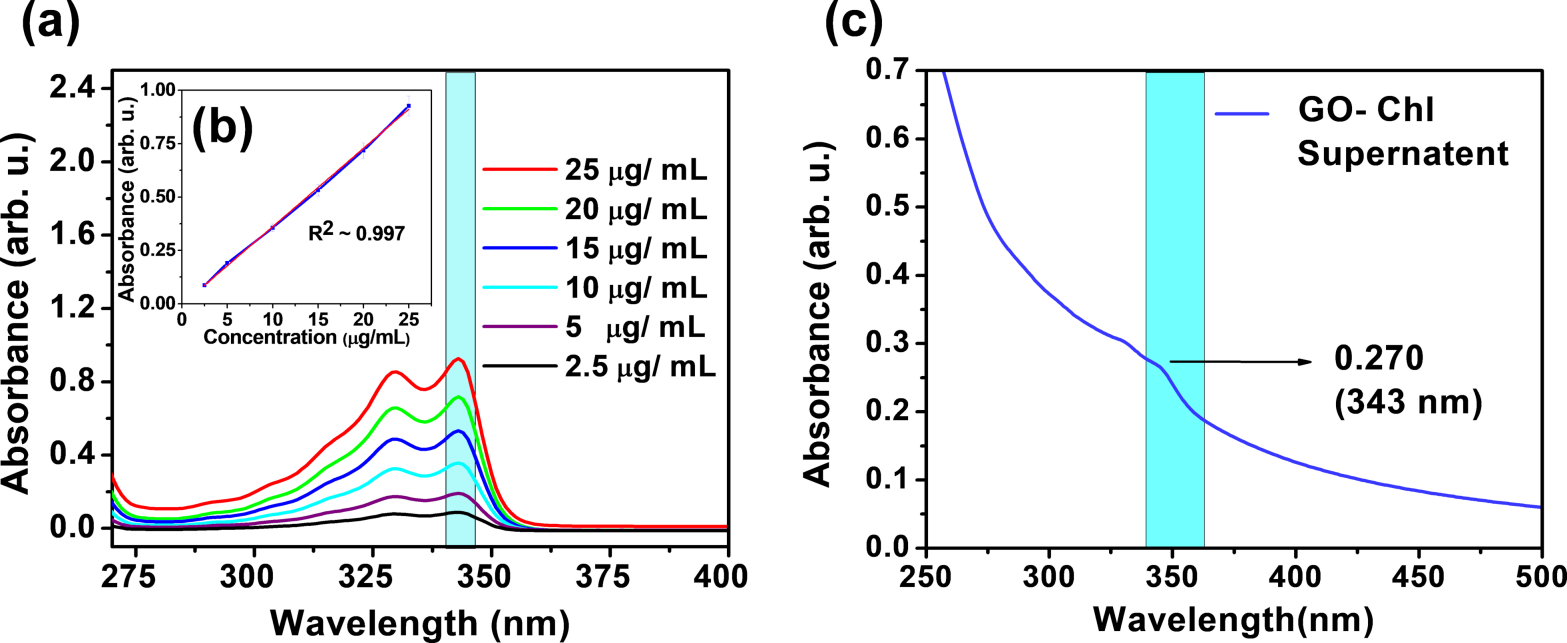
Quantitative estimation of Chl binding onto GO nanosheets using UV–vis spectroscopy: (a) Optical density measurement of various concentrations (2.5–25 µg/mL) of Chl for the preparation of a standard calibration curve. (b) Linearity plot (*R*^2^ ≈ 0.997) prepared by measuring the relative optical density for different concentrations of Chl, and (c) optical density measurement of the supernatant having unattached Chl.

The amount of Chl bound onto GO sheets was estimated in two ways viz. entrapment efficacy (EE, % Chl that has been successfully absorbed onto GO) and % drug content or drug loading efficiency (DLE, amount of Chl loaded per unit weight of GO) using UV–vis spectroscopy. Firstly, a standard calibration curve of Chl was plotted by monitoring the optical density at 343 nm (as shown in Figure 3a) and the concentration of Chl present in the GO–Chl nanoconjugate was calculated using the calibration curve. The values for EE and DLE were calculated using Equation 1 and 2 and the results are summarized in Table 1.


[1] $$\text{Entrapment Efficiency}\left(\text{EE}\right)=\frac{\left(A-B\right)}{A}\times 100,$$

where *A* and *B* are the total amount of drug used in 115 mL of solution and the amount of unbound drug left in supernatant, respectively.


[2] $$\text{Drug Loading Efficiency}\left(\text{DLE}\right)=\frac{\left(m_{\text{o}}-m_{\sup}\right)}{m_{\text{GO}}}\times 100\%,$$

where *m*_o_, *m*_sup_, and *m*_GO_ are total amount of drug used initially, unbound drug obtained in the supernatant, and weight of GO, respectively.

**Table 1:** Estimation of entrapment efficacy and drug loading efficiency using UV–visible spectroscopy.

S. NO.	Total amount of Chl used in 115 mL (A)	Total amount of Chl per mL	OD of supernatant	Amount of drug left in supernatant (From OD) (B)	EE = 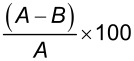	DLE = 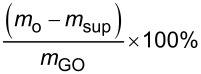

1	7500 μg in 115 mL	65.217 μg/mL	0.270	7.5 μg/mL	88.49	6.637%

### Cellular internalization of the GO–Chl nanoconjugate and flow-cytometry-based propidium iodide uptake analysis

**Figure 4:**
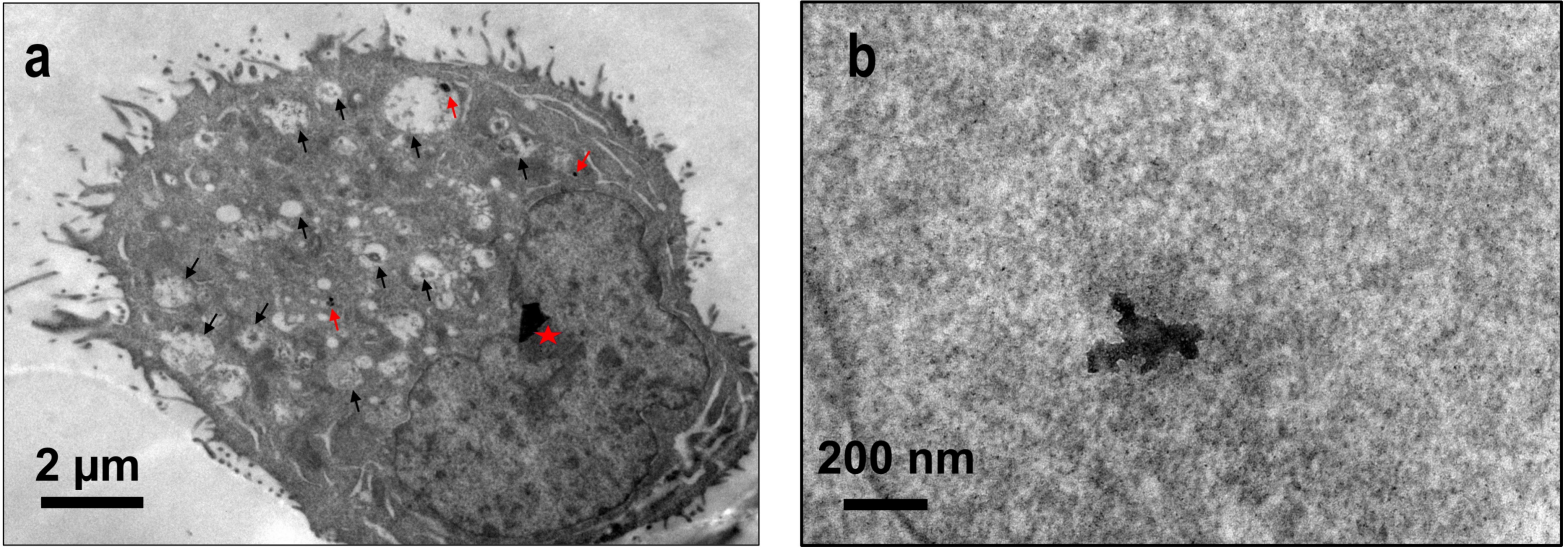
Analysis of cellular internalization of GO–Chl nanoconjugates in A549 cells: (a) Transmission electron micrographs showing the presence of vacuoles (black arrows), nanoconjugate uptake, and presence in vacuoles (red arrows). The red star denotes an artifact. (b) Presence of GO–Chl nanoconjugate in A549 lung cancer cells at a higher magnification.

The efficacy of nanomedicines mainly depends upon the effective cellular internalization and their transport to the appropriate intercellular effector site [52–53]. Studies have shown that based on its size and surface characteristics (i.e., hydrophilicity or hydrophobicity, and C/O ratio) GO is internalized via clathrin or caveolae-mediated endocytosis and micropinocytosis [53]. The exposure to nanomaterials is known to affect plasma membrane integrity, which in turn initiates various metabolic processes, such as ineffective nutrient transport, unspecific molecular targeting, DNA damage, and dysfunction of other intracellular organelles [52,54]. To assess the interaction of GO–Chl with A549 cells, we incubated the cells with 25 µg/mL of GO–Chl for 24 h at 37 °C and processed the samples as described in the method section. After 24 h of incubation, an increased number of vacuoles were observed in the treated cells (Figure 4a, black arrows). Furthermore, TEM micrographs reveal the presence and effective cellular internalization of the GO–Chl nanoconjugate in A549 cells (Figure 4a; red arrows and Figure 4b), corroborating our previous findings [25].

**Figure 5:**
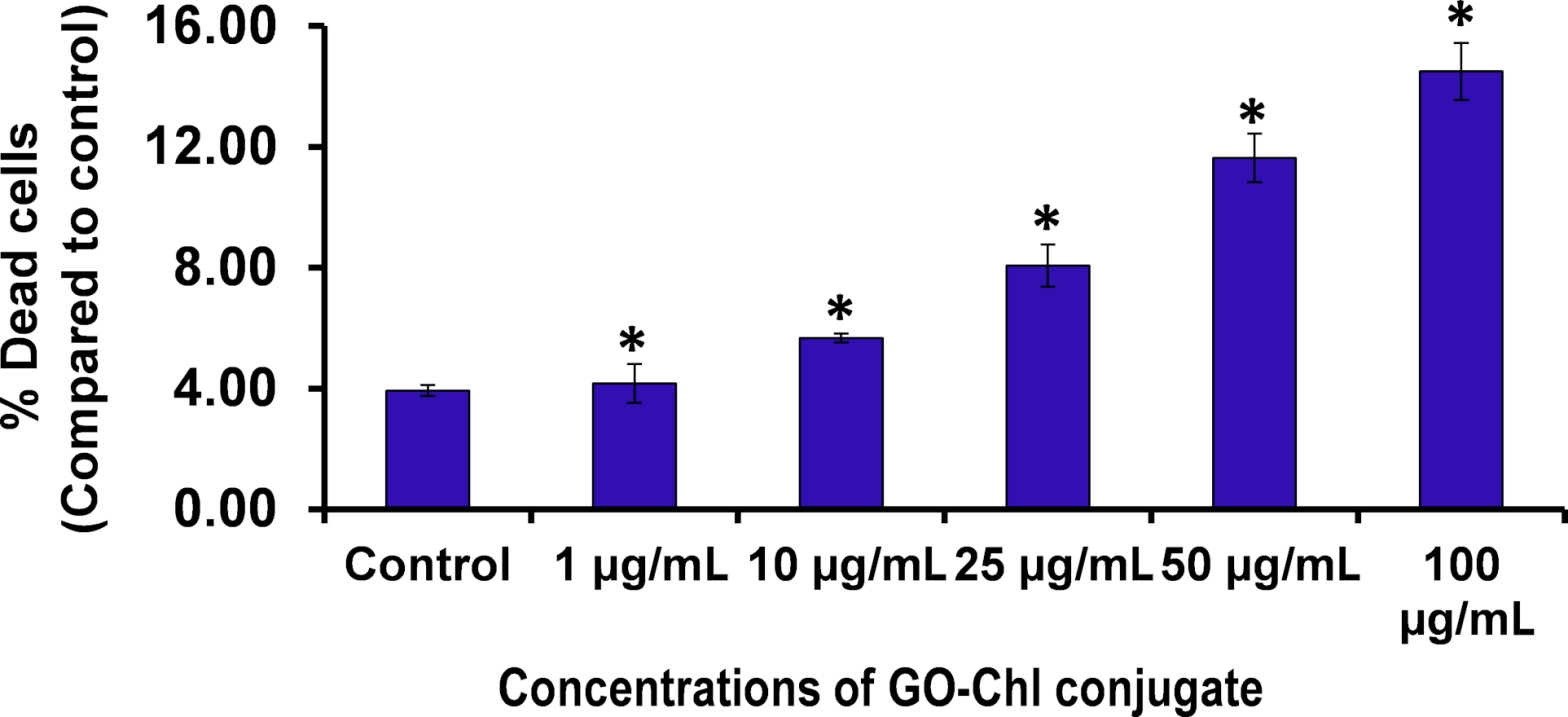
Flow-cytometry-based propidium iodide uptake analysis of A549 cells exposed to GO–Chl: A dose-dependent increase in the concentration of cells with compromised cell membranes was observed. Values are expressed as mean ± SEM of three independent experiments. A value of *p* < 0.05 (*) was considered statistically significant.

To investigate the effect of GO–Chl on plasma membrane integrity and cell viability, we performed flow-cytometry-based PI uptake analyses. Figure 5 reveals the dose-dependent increase in the number of cells with compromised membranes, which indicates significant growth in the number of dead A549 cells after exposure to GO–Chl. These results are in good agreement with our previously reported MTT-based cell death data of GO–Chl-exposed A549 lung cancer cells [25].

### Cell cycle progression analysis using flow-cytometry-based propidium iodide assay

**Figure 6:**
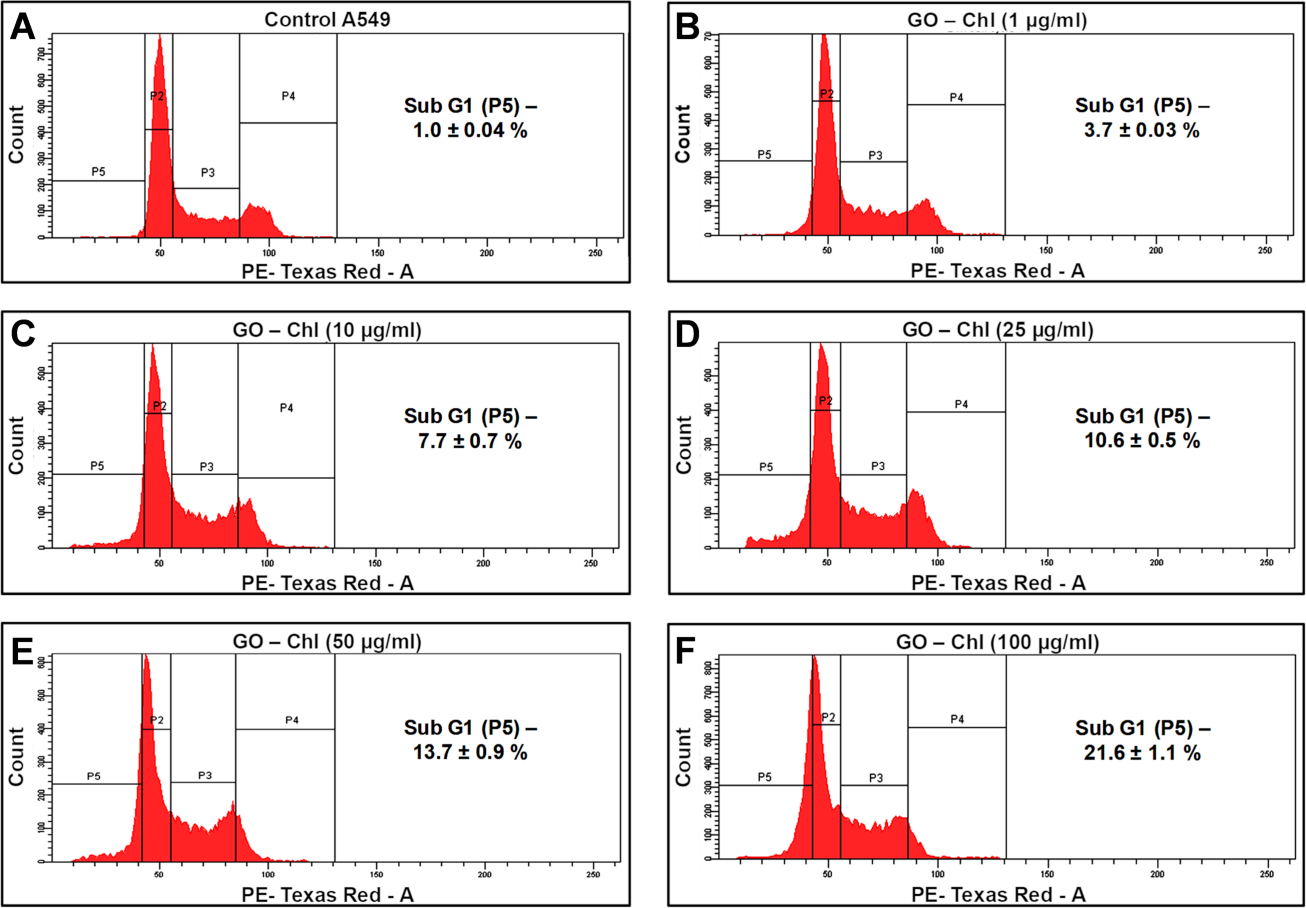
Flow-cytometry-based cell cycle analysis of A549 cells exposed to GO–Chl. Different gates were used to represent the cells in various phases of the cell cycle: P2 represents the G1 phase, P3 represents the S phase, P4 represents the G2 phase, and P5 represents the subG1 phase. Values are expressed as mean ± SEM of three independent experiments. A value of *p* < 0.05 (*) was considered statistically significant.

The disruption of plasma membrane integrity triggers enhanced permeability to chemotherapeutics, which leads to alterations in the cell cycle, genotoxicity, and autophagy modulation in cancer cells [39,55]. The cell cycle includes a division phase and an interphase which are responsible for the efficient cellular physiology and metabolic pathways. Any impairment in the cell cycle could be used as a measure of genotoxic alterations induced by any external stresses [56]. The cell cycle involves a series of highly regulated events for cell growth, DNA replication, and cell division to produce daughter cells. Graphene oxide has been found to interfere with DNA replication genes and causes alterations in the cell cycle, mutagenesis, and elevated expression of genes that mediate DNA-damage control and apoptosis in cancer cells [55]. Therefore, to assess the effect of GO–Chl on the cell cycle process in A549 cells, we performed flow-cytometry-based cell cycle analysis. Figure 6 reveals the cell cycle analysis of A549 cells exposed to GO–Chl. Results showed a significant increase in the population of cells in the sub G1 phase (reduced DNA content), which could be attributed to possible DNA fragmentation and ultimate A549 cell death by the exposure to the GO–Chl nanoconjugate. However, as observed in Figure 6E and Figure 6F, the P2 population was slightly moved towards the P5 which could be attributed to either the arrest of cells in the subG0/G1 phase before their migration or could be related to alterations in the marker intensity due to the treatments.

### Genotoxicity assessment in GO–Chl-exposed A549 lung cancer cells using single-cell gel electrophoresis

**Figure 7:**
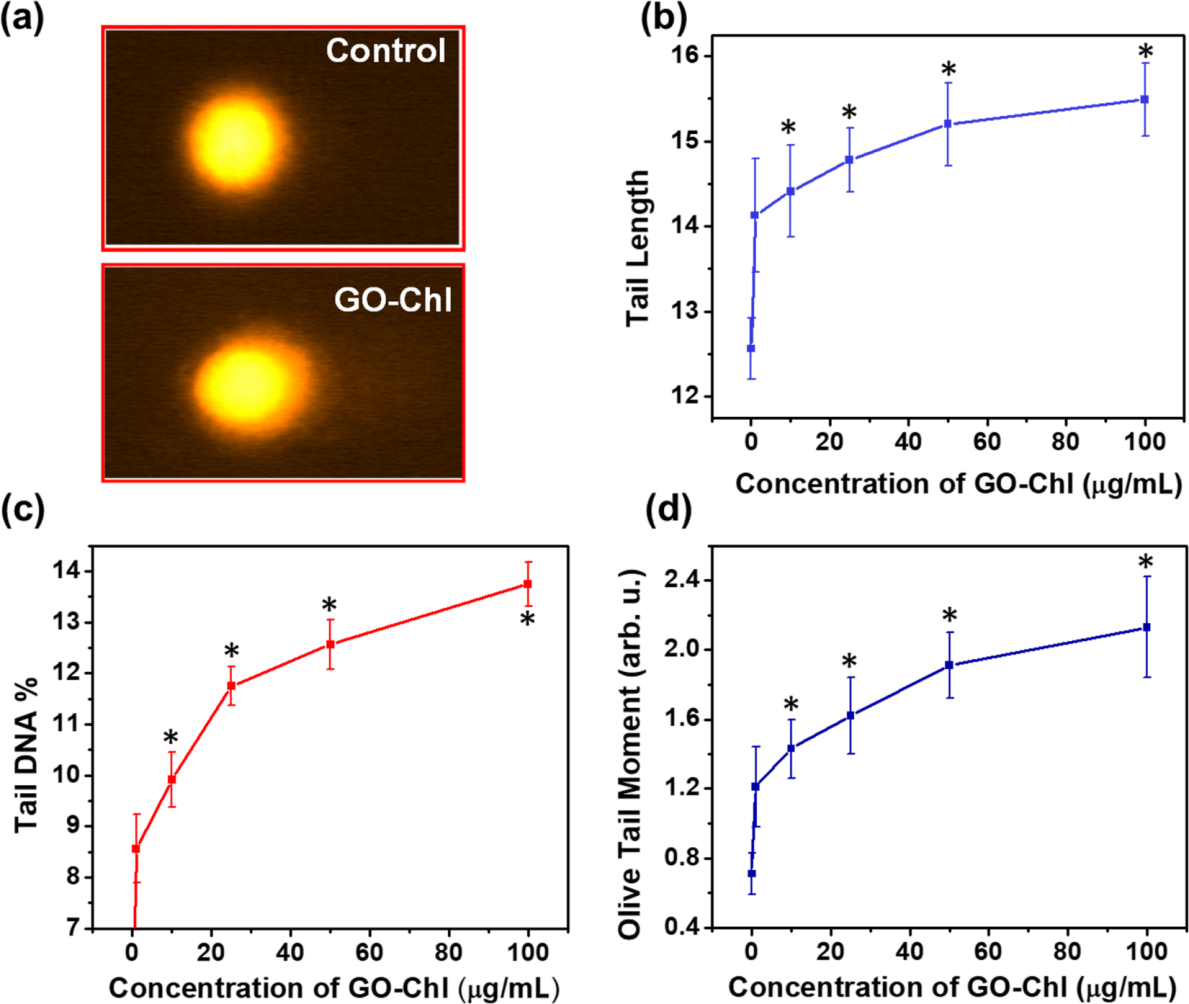
Comet assay of GO–Chl nanoconjugates in A549 lung cancer cells: (a) digital image of the comet formation, (b–d) concentration-dependent analysis of tail length, tail % DNA, and Olive tail moment upon exposure of A549 lung cancer cells to GO–Chl. Values are expressed as mean ± SEM of three independent experiments. A value of *p* < 0.05 (*) was considered statistically significant.

To investigate the genotoxicity and DNA damage induced in GO–Chl-exposed A549 lung cancer cells, we performed single-cell gel electrophoresis, or comet assay, which is a versatile and sensitive technique to measure single/double strand DNA breaks, DNA crosslinks, base damages, and apoptotic nuclei [57]. Figure 7 reveals the DNA damage analysis in GO–Chl-exposed A549 cells. In Figure 7a, the digital images of DNA comets obtained by florescence microscopy indicates that GO–Chl exposure induces a strong genotoxic effect in A549 cells. Furthermore, a concentration-dependent increase in the tail length was observed (Figure 7b), which is a measure of the extent of DNA damage by analyzing the length of DNA migration during electrophoresis. Furthermore, statistical analysis of the resulting tail-length data yielded tail DNA percentage and Olive tail moment using Equation 3 and Equation 4, respectively.


[3] $$\text{\%DNA in tail}=\frac{\text{total intensity of tail}}{\text{total intensity of comet}}\times 100$$

[4] Tail Moment=Tail length×%DNA in tail

Figure 7b–d reveal a dose-dependent increase in tail length, tail DNA percentage, and Olive tail moment, and collectively show the genotoxicity induced in GO–Chl-exposed A549 cells. Recently, it has been shown that exposure to higher concentrations of GO could induce DNA damage through the base excision repair (BER) pathway in HEK 293T cells [58]. The presence of high mobility and sharp edges of GO could potentially contribute to the genotoxic behavior [58]. On the other hand, Chl have shown its capacity for inducing genotoxicity in cancer cells in a ROS-dependent manner [59]. The results indicate that induction of DNA damage in GO–Chl-exposed A549 lung cancer cells could possibly be due to alteration in/failure of DNA-damage response mechanisms.

### Role of autophagy machinery in inducing DNA damage in GO–Chl-exposed A549 lung cancer cells

Cells upon exposure to external stress and endogenous metabolic changes produce a variety of DNA lesions. Those can give rise to genomic instability via gene mutations and chromosomal damage, leading to tumor progression and metastasis [60]. In a healthy cell, various DNA-damage response pathways and DNA-repair proteins get activated to overcome the genetic modification caused by DNA lesions and determining the fate of cell survival and cell death [60]. In response to a particular type of DNA damage, highly selective and complex cell signaling networks, such as the BER pathway (for SSBs), homologous recombination repair and non-homologous end-joining pathways (for DSBs), nucleotide excision repair pathways (for bulky adducts formations), and mismatch repair pathways (for nucleotide mutations) get activated to ensure cell survival [61]. During an early stage of tumorigenesis, the deregulation of cell proliferation results in loss of one or more DNA-damage pathways in cancer cells, leading to greater dependency on DDR. Therefore, the tendency of a cancer cell to harbor DDR dependency through activation of complex molecular signaling pathways, such as poly-ADP ribose polymerase (PARP-1), DNA-dependent protein kinase (DNA-PK), and activation of the autophagy machinery could provide multiple target therapeutic windows to treat patients with tumors lacking specific DDR functions [60,62].

**Figure 8:**
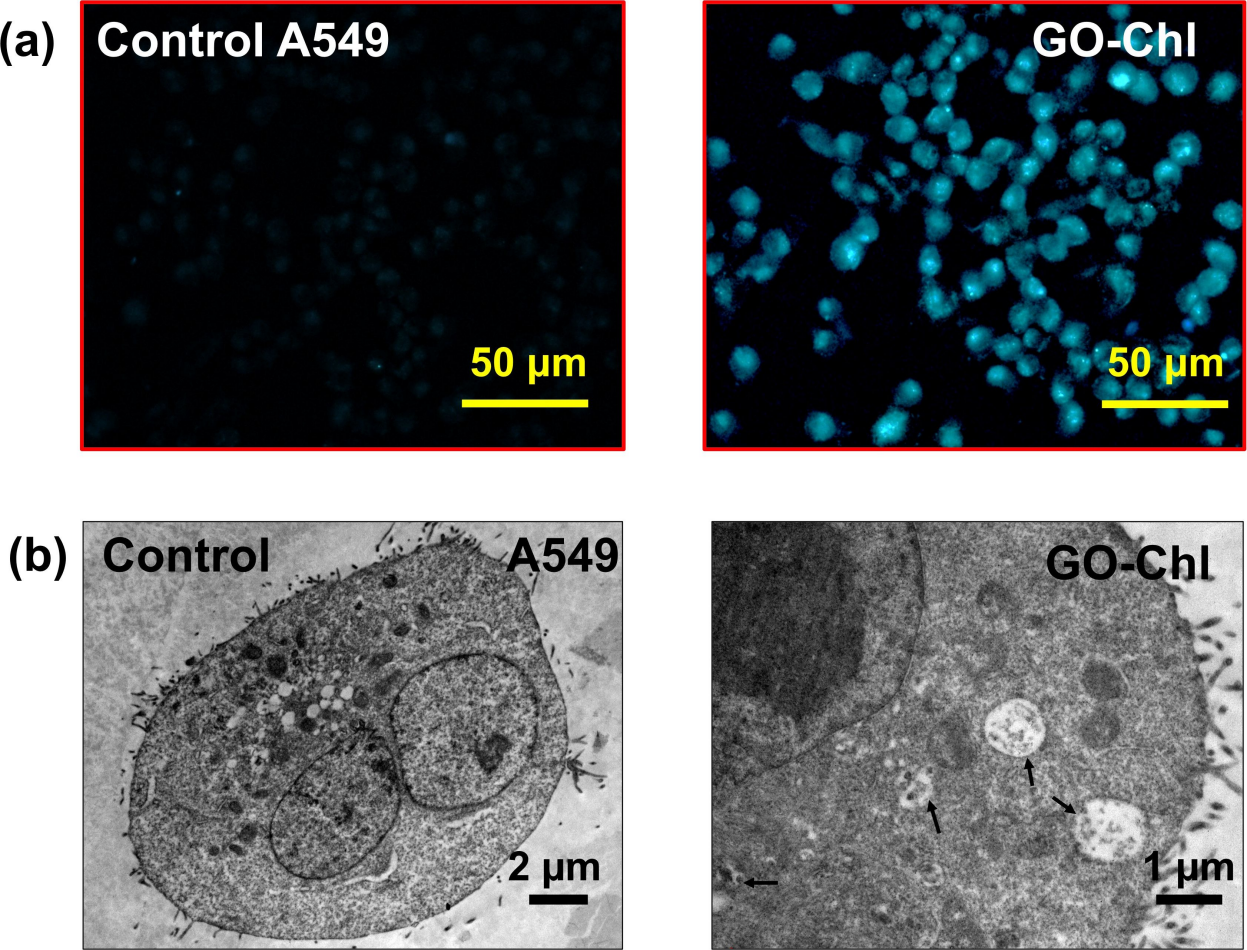
Accumulation of autophagosomes in GO–Chl-exposed A549 lung cancer cells: (a) Representative monodansylcadaverine staining photomicrographs of A549 cells exposed to GO–Chl (25 μg/mL) for the determination of acidic vacuoles (scale bar = 50 μm). (b) Representative transmission electron microscopy photomicrographs of GO–Chl-(25 μg/mL) exposed A549 cells showing the presence of autophagosomes. Black arrows indicate the presence of autophagosomes. Scale bars are 2 µm (left image) and 1 µm (right image), respectively.

In response to DNA damage and chromosomal aberrations, multidrug-resistant tumors activate the autophagy machinery as a survival mechanism [63]. However, accumulating evidence supports the fact that inhibition of autophagy by exposure to either nanomaterials or pharmacological inhibitors could potentially inhibit the activation of DNA-damage response mechanisms in different cancer cells and animal models [23,64]. To elucidate the role of autophagy in DNA damage in GO–Chl-exposed A549 cells, we performed a set of experiments focusing on autophagosomes. Figure 8a reveals the florescence-microscopy-based MDC staining for labeling autophagic vacuoles in GO–Chl-exposed A549 cells. The results reveal a significantly high number of acidic vacuoles (MDC positive) in GO–Chl-exposed cells as compared to control cells after 24 h of exposure to GO–Chl (25 µg/mL). The MDC dye selectively accumulates in autophagosomes or other acidic cellular vacuoles [34]. Therefore, we used it to confirm the presence of autophagosomes via TEM. Figure 8b reveals the appearance of autophagosomes in GO–Chl-exposed A549 cells. The MDC staining assay and TEM analysis together show the appearance of autophagosomes, which could be due to inhibition of autophagy.

**Figure 9:**
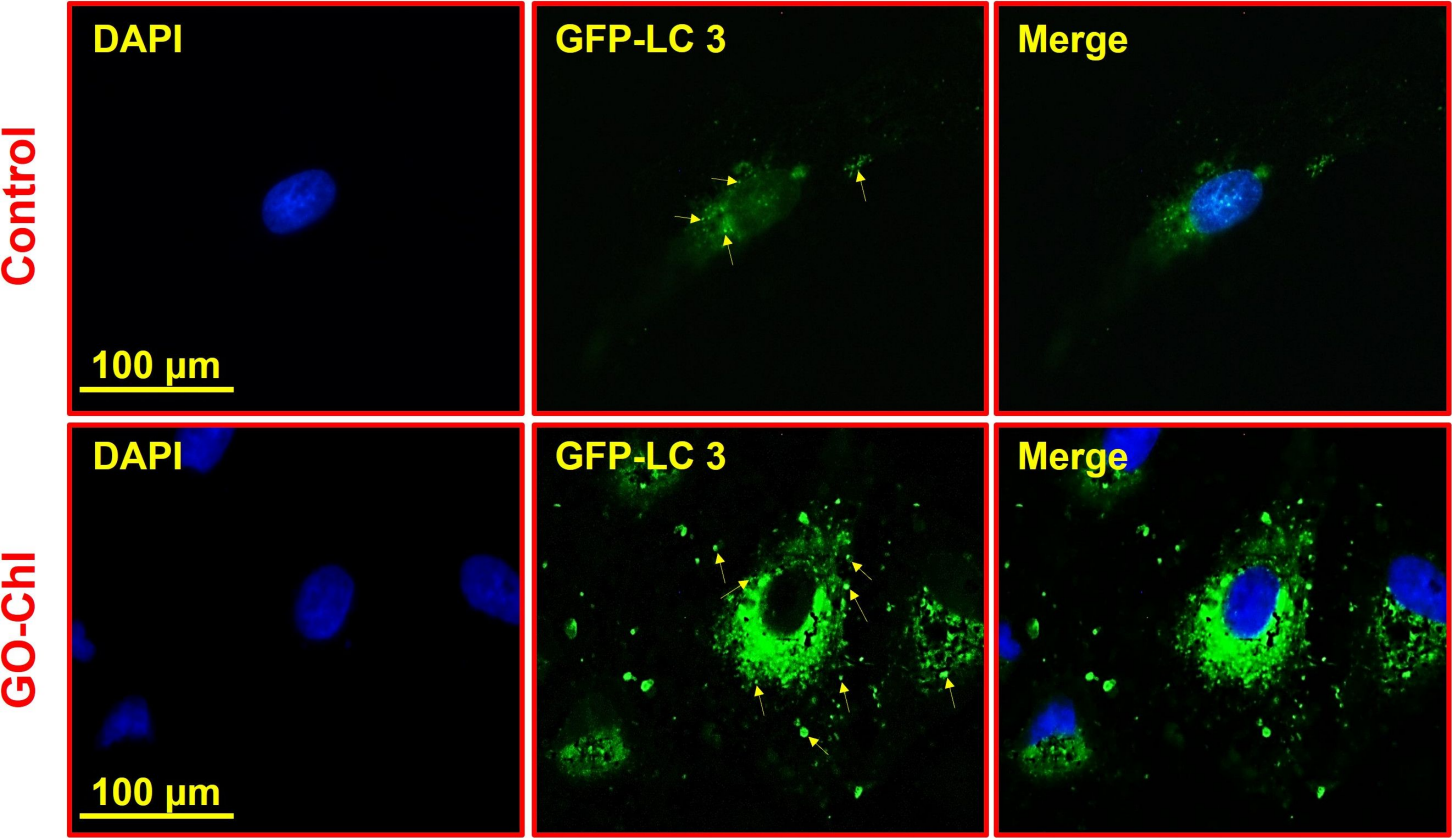
Mammalian GFP-LC3 transfection assay for autophagic flux analysis in A549 cells exposed to GO–Chl nanoconjugates. Cells were transfected with the GFP-LC3 plasmid for six hours followed by treatment with 25 µg/mL of GO–Chl for 24 hours. Representative photomicrographs showing the increased accumulation of GFP-LC3 in GO–Chl-exposed A549 cells compared to control cells. Scale bar 100 µm.

Furthermore, we performed confocal microscopy with GFP-LC3 plasmid-transfected cells to investigate and quantify the accumulation of autophagosomes upon GO–Chl exposure. Mammalian LC3 is a specific marker to monitor autophagy through its incorporation into the autophagosomal membranes. During the course of autophagy, the fusion of the autophagosome with lysosomes results in a very low LC3 content in autolysosomes due to subsequent degradation of LC3 by lysosomal enzymes [65]. Therefore, the endogenous GFP-LC3 is visualized as a diffuse cytoplasmic pool or punctate structures and could be used for the selective quantification of autophagosomes, thus allowing the monitoring of autophagy inhibition [66]. Figure 9 shows a significant increase in the number of LC3 punctate and confirms the accumulation of autophagosomes in GO–Chl-exposed A549 cells.

**Figure 10:**
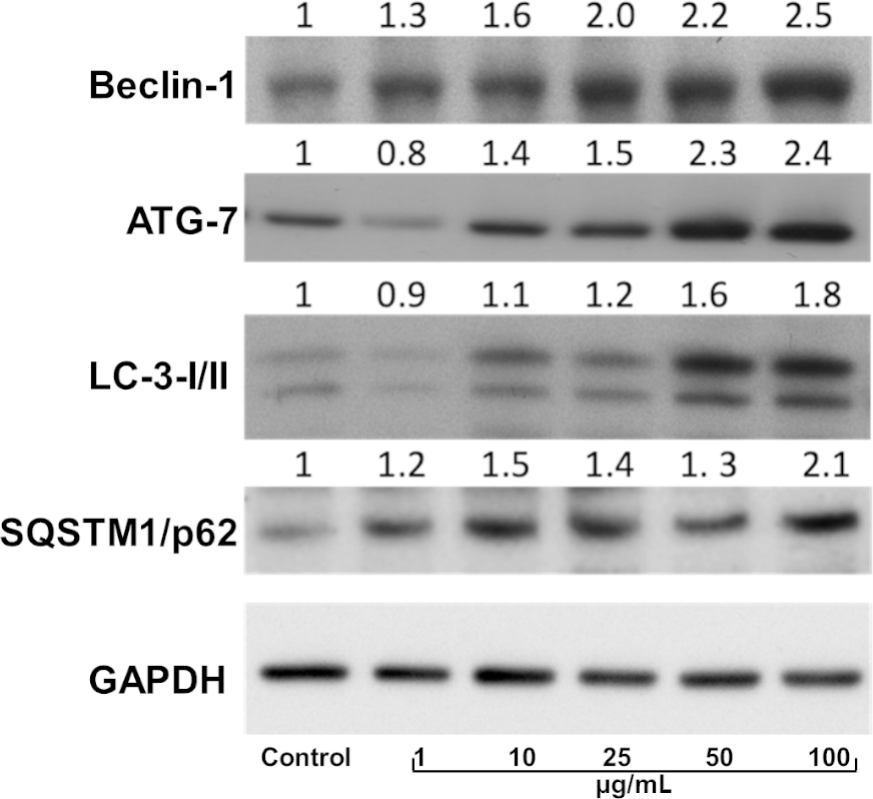
Western blot analysis of autophagy markers in cell lysates of GO–Chl-exposed A549 cells in a dose-(μg/mL) dependent manner. A549 cells were exposed to various concentrations of of GO–Chl (1–100 µg/mL) for 24 hours and the whole protein was extracted using RIPA buffer. The values are expressed as mean ± SEM of three independent experiments.

**Figure 11:**
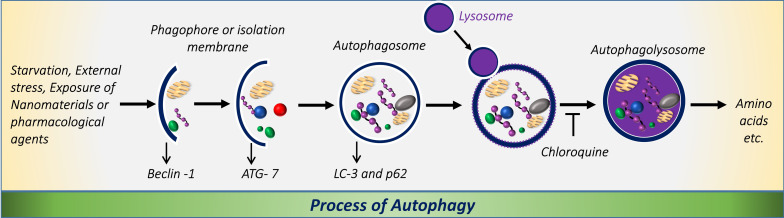
Schematic showing the process of autophagy in mammalian cells, and the modulation of autophagy by chloroquine by inhibiting the fusion between the autophagosome and the lysosome.

Autophagy requires the regulation of a number of key proteins, such as Rad51, BLM/WRN, DNA helicases, the Mre11 complex, or TopBP1, and DNA repairing enzymes (e.g., DNA-PKcs and MGMT), responsible for DNA-damage associated cell death [67]. Recent studies have shown that inhibition of autophagy at different stages caused variation in the expression level of key autophagy proteins, such as beclin-1, ATG-7, LC-3-I/II, and SQSTM1/p62, which could regulate the DNA-damage response in cancer cells [67]. Therefore, to investigate the molecular mechanism responsible for inducing DNA damage through autophagy modulation in A549 cells upon GO–Chl nanoconjugate exposure, we performed immunoblot analysis of various related proteins. Figure 10 reveals the expression level of key autophagy proteins responsible for the formation of autophagosomes and functioning of the autophagy process. Figure 11 schematically represents the involvement of key proteins in different steps of the autophagy process in mammalian cells. A significant increase in the expression of beclin-1 and ATG-7 was observed in GO–Chl-exposed A549 cells. Beclin-1 is a three-structure-domain protein (BH-3 only, CCD, and ECD) and is key in the regulation of autophagy in mammalian cells [68]. Recent studies reveal that elevated expression levels of beclin-1 leads to enhanced DNA damage in cancer cells, leading to increase in cell death [69]. On the other hand, ATG-7 is a key protein in the formation of the autophagosome through phagophore membrane elongation via activation of a ubiquitin-like conjugation system (ATG5-ATG12-ATG16L1) [70]. The high expression of the ATG-7 protein observed points towards the formation of autophagosomes in GO–Chl-exposed A549 cells, and corroborates the results obtained through MDC staining and confocal microscopy analysis.

Furthermore, a significant dose-dependent increase in the expression level of LC-3 II proteins was observed. LC-3 is a microtubule-associated protein 1A/1B – light chain 3 (LC-3) that becomes lipidated and tightly associated with the autophagosome membrane [71]. LC-3 proteins are incorporated onto the autophagosome membrane, which is later degraded by autolysosomes (formed by the fusion between autophagosomes and lysosomes) [72]. Elevated expression of LC-3-I/II biomarkers indicates the formation and accumulation of autophagosomes, and suggests the modulation of the autophagy process through impaired autophagic flux partly caused by chloroquine in GO–Chl-exposed A549 cells.

In addition, a significant increase in the expression levels of SQSTM1/p62 was observed, demonstrating the modulation of autophagy at a later stage by inhibiting the fusion of autophagosome with lysosomes. SQSTM1/p62 is a multi-domain protein that interacts with the autophagy machinery to regulate various cellular metabolic processes [73]. Recent studies reveal the importance of p62 in regulating cell death processes, harnessing the DNA-damage response capability, and inducing complex signaling networks responsible for cellular detoxification [73–74]. Most importantly, it was found that the autophagy substrate SQSTM1/p62 inhibits the E3 ligase RNF168-dependent ubiquitination of chromatin, and plays a crucial role in the dysfunction of DNA repair capacity through autophagy modulation [22]. The elevated expression of p62 observed in the present study is in good agreement with the previously reported data, suggesting the critical role of the autophagy machinery in harnessing the DNA-damage response in GO–Chl-exposed A549 cells. However, a detailed mechanistic investigation of the key check points will be required. Therefore, from the available literature and experimental observations from the present study, it becomes clear that the autophagy machinery plays an important role in regulating the DNA-damage response in A549 cells, and that the GO–Chl nanoconjugate can induce the DNA damage through SQSTM1/p62-mediated autophagy modulation.

## Conclusion

We have successfully demonstrated the genotoxicity induced in A549 lung cancer cells by exposure to the GO–Chl nanoconjugate and clarified the role of autophagy modulation in harnessing the DNA-damage response. Flow-cytometry-based PI uptake reveals a significant loss of plasma membrane integrity, which leads to cell cycle arrest. Furthermore, single-cell gel electrophoresis (or comet assay) reveals a significant dose-dependent increase in tail length, tail DNA percentage, and Olive tail moment as a measurement of genotoxicity in GO–Chl-exposed A549 cells.

To access the role of autophagy modulation by the GO–Chl nanoconjugate, we have employed MDC staining, confocal microscopy using GFP-LC3 transfected cells, and TEM analysis. A significant dose-dependent increase accumulation of autophagosomes was observed, suggesting inhibition of autophagy and the possible connection between DNA-damage response and autophagy. Finally, elevated expression levels of key autophagy proteins such as beclin-1, ATG-7, LC-3-I/II, and SQSTM1/p62 reveal that inhibition of autophagy at later stages plays a crucial role in regulating the DNA-damage response capability in GO–Chl-exposed A549 cells. These results reveal the efficacy of the GO–Chl nanoconjugate as a nanodrug candidate alone or possibly in combination with other chemotherapeutic drugs, which require further investigation, through unique DNA damage/autophagy synergy.

## Supporting Information

The file contains three figures and one table. Figure S1 demonstrates the optical, functional, structural, and morphological analysis of GO and GO–Chl nanoconjugate via UV–vis, FTIR, Raman Spectroscopy, FESEM, and HRTEM. Figure S2 shows the atomic force microscopy-based topographical analysis of GO nanosheets. Figure S3 represents the XPS survey spectra of GO, GO–Chl, and Chl. Table S1 shows the summary of fitting parameters for the C1s core level spectra for GO, Chl, and GO–Chl samples.

File 1 Additional figures (S1–S3) and table (S1).

## Data Availability

All data that supports the findings of this study is available in the published article and/or the supporting information of this article.
