# Supplementary material for: Graphene oxide–chloroquine conjugate induces DNA damage in A549 lung cancer cells through autophagy modulation
Source: Beilstein J Nanotechnol. 2025 Mar 3;16:316–32. doi: 10.3762/bjnano.16.24 (PMC11878127; doi:10.3762/bjnano.16.24)
Supplement: File 1 — Additional figures (S1–S3) and table (S1). [file Beilstein_J_Nanotechnol-16-316-s001.pdf]

BEILSTEIN JOURNAL OF NANOTECHNOLOGY

Supporting Information

for

Graphene oxide–chloroquine conjugate induces DNA damage in A549 lung cancer cells through autophagy modulation

Braham Dutt Arya, Sandeep Mittal, Prachi Joshi, Alok Kumar Pandey, Jaime E. Ramirez-Vick, Govind Gupta and Surinder P. Singh

Beilstein J. Nanotechnol. **2025,** *16,* 316–332. doi:10.3762/bjnano.16.24

Additional figures (S1–S3) and table (S1)

License and Terms: This is a supporting information file under the terms of the Creative Commons Attribution License (https://creativecommons.org/licenses/by/4.0). Please note that the reuse, redistribution and reproduction in particular requires that the author(s) and source are credited and that individual graphics may be subject to special legal provisions.
The license is subject to the *Beilstein Journal of Nanotechnology* terms and conditions: (https://www.beilstein-journals.org/bjnano/terms)

Supplementary Figure 1:

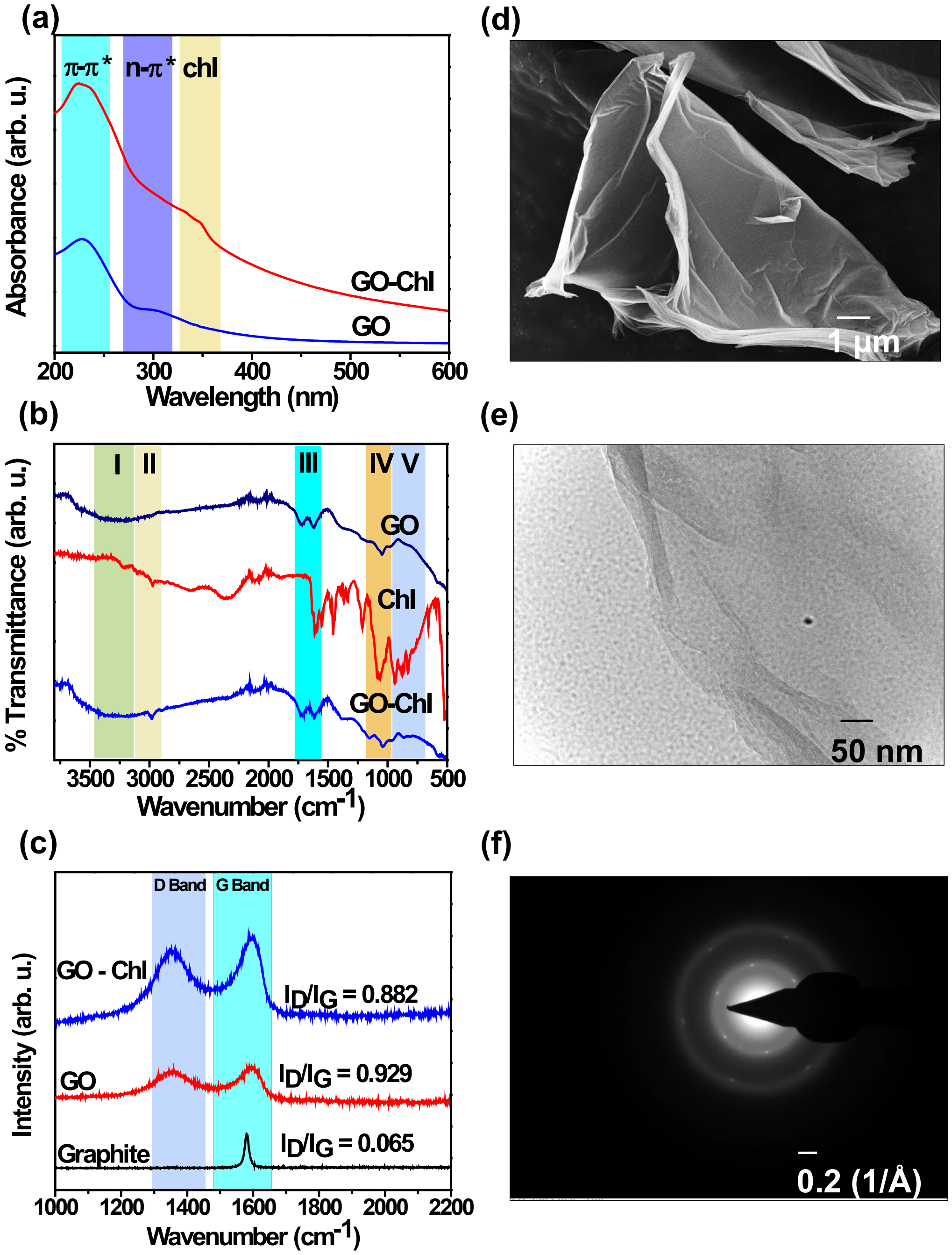

Figure S1: Optical, functional, structural, and morphological analysis of GO and GO–Chl nanoconjugate: (a) UV–vis spectra of GO and GO–Chl. (b) Fourier-transform infrared spectra of GO, Chl, and GO–Chl. (c) Raman spectra of graphite, GO, and GO–Chl. (d) Field-emission scanning electron microscopy photomicrograph. (e) High-resolution transmission electron microscopy image of GO and (f) selected area of the electron diffraction pattern of GO.

Supplementary Figure 2:

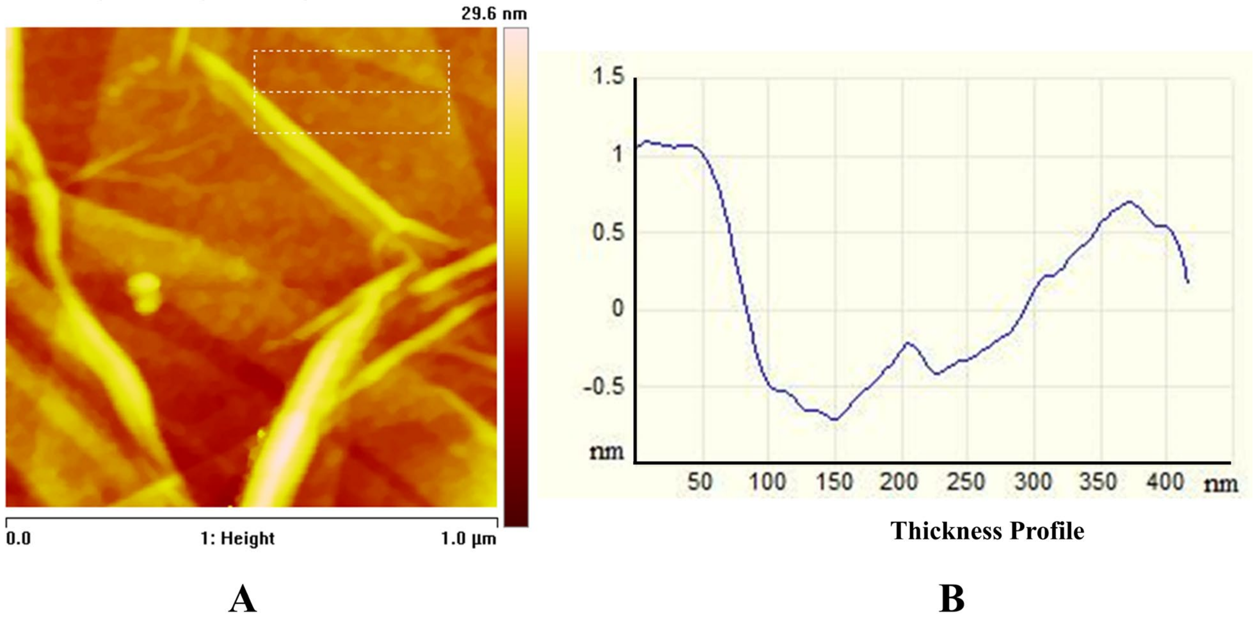

Figure S2: A) Atomic force microscopy based topographical analysis of GO nanosheets casted on a silicon wafer substrate (scale 0–1 µm). B) Height and thickness profile of GO nanosheets obtained through AFM.

Supplementary Figure 3:

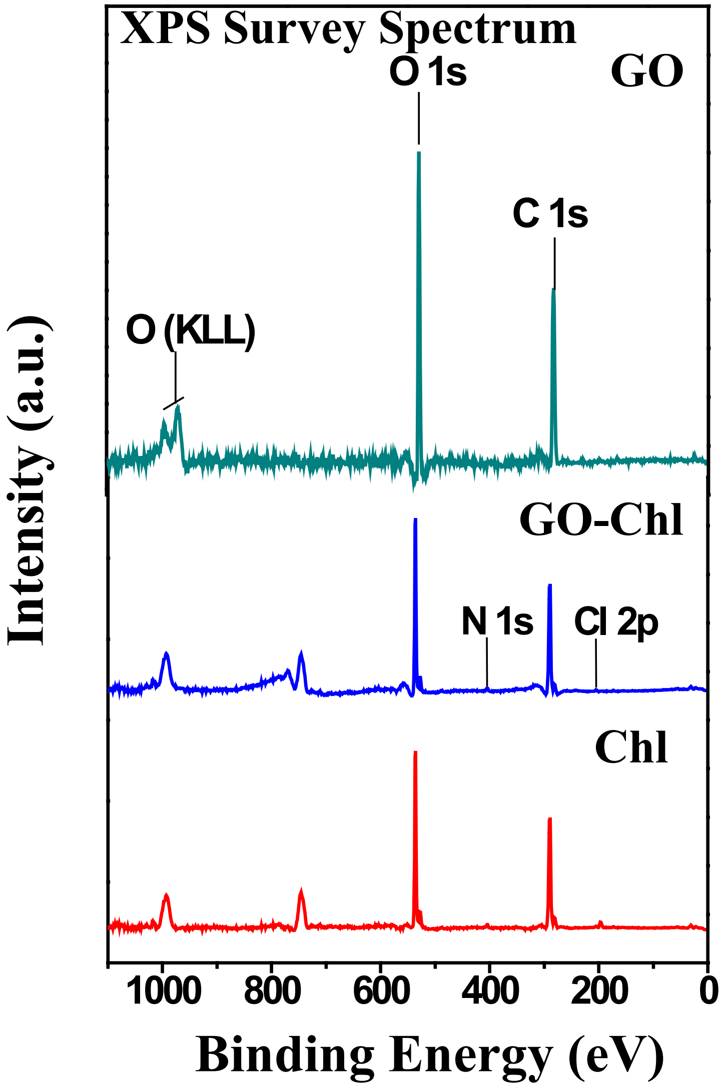

Figure S3: XPS survey spectra of GO, GO–Chl, and Chl.

Table S1: Summary of fitting parameters for the C 1s core level spectra for GO, Chl, and GO–Chl samples. Uncertainty in determining the binding energy (B.E.) position and full width at half maximum (FWHM) is estimated to be ±0.05 eV. The uncertainty in determining the relative percentage is estimated to be ±5 % of the base value.

	**GO**			**Chl**			**GO-Chl**		
**Peak**	**B.E. (eV)**	**FWHM (eV)**	**Relative %**	**B.E. (eV)**	**FWHM (eV)**	**Relative %**	**B.E. (eV)**	**FWHM (eV)**	**Relative %**
**C–C**				284.6	1.65	11.93	284.6	1.63	13.85
**C=C**	284.4	1.62	7.27						
**C–N**				285.6	1.63	13.64	285.6	1.63	38.13
**C–OH**	285.6	1.63	20.09						
**C–Cl**				286.4	1.41	42.69	286.4	1.38	11.88
**C–O**	286.6	1.38	20.83						
**C=N**				287.6	1.41	31.73	287.6	1.43	26.79
**C=O**	287.7	1.48	21.54						
**COOH**	288.8	1.73	30.25				288.9	1.88	9.35
